# Diversity and Functional Evolution of Terpene Synthases in Dictyostelid Social Amoebae

**DOI:** 10.1038/s41598-018-32639-0

**Published:** 2018-09-25

**Authors:** Xinlu Chen, Tobias G. Köllner, Gad Shaulsky, Qidong Jia, Jeroen S. Dickschat, Jonathan Gershenzon, Feng Chen

**Affiliations:** 1 Department of Plant Sciences, University of Tennessee, Knoxville, TN 37996 USA; 2 Department of Biochemistry, Max Planck Institute for Chemical Ecology, Jena, Germany; 3 Department of Molecular and Human Genetics, Baylor College of Medicine, Houston, TX 77030 USA; 4 Kekulé-Institute of Organic Chemistry and Biochemistry, University of Bonn, Gerhard-Domagk-Straße 1, 53121 Bonn, Germany

## Abstract

Dictyostelids, or social amoebae, have a unique life style in forming multicellular fruiting bodies from unicellular amoeboids upon starvation. Recently, dictyostelids were found to contain terpene synthase (TPS) genes, a gene type of secondary metabolism previously known to occur only in plants, fungi and bacteria. Here we report an evolutionary functional study of dictyostelid *TPS* genes. The number of *TPS* genes in six species of dictyostelids examined ranges from 1 to 19; and the model species *Dictyostelium purpureum* contains 12 genes. Using *in vitro* enzyme assays, the 12 *TPS* genes from *D*. *purpureum* were shown to encode functional enzymes with distinct product profiles. The expression of the 12 *TPS* genes in *D*. *purpureum* is developmentally regulated. During multicellular development, *D*. *purpureum* releases a mixture of volatile terpenes dominated by sesquiterpenes that are the *in vitro* products of a subset of the 12 *TPS* genes. The quality and quantity of the terpenes released from *D*. *purpureum*, however, bear little resemblance to those of *D*. *discoideum*, a closely related dictyostelid. Despite these variations, the conserved clade of dictyostelid TPSs, which have an evolutionary distance of more than 600 million years, has the same biochemical function, catalyzing the formation of a sesquiterpene protoillud-7-ene. Taken together, our results indicate that the dynamic evolution of dictyostelid *TPS* genes includes both purifying selection of an orthologous group and species-specific expansion with functional divergence. Consequently, the terpenes produced by these TPSs most likely have conserved as well as species-adaptive biological functions as chemical languages in dictyostelids.

## Introduction

Dictyostelids, known as social amoebae, are soil-dwelling eukaryotes that have a unique life style. When food (bacteria) is abundant, dictyostelids grow vegetatively as free-living, unicellular amoeboid cells through cell division. On the contrary, when food is depleted, dictyostelids form multicellular fruiting bodies by the aggregation of unicellular amoeboid cells. The transition from the unicellular form to fruiting bodies is a tightly regulated developmental process^1^. With the aid of genome sequencing and comparative genomics, genes underlying conditional multicellularity in dictyostelids continue to be identified and characterized^2–4^. Among the many features of the genome sequences of dictyostelids is the presence of notable types and numbers of genes of secondary metabolism^5,6^, among which polyketide synthase genes are the best recognized^7^. Forming a large gene family in each species, polyketide synthase genes exhibit extensive species-specific expansion^6,8^ and have been suggested to synthesize various types of polyketides for signaling and defense^6,9^. Along this line, an important recent finding is that dictyostelids also contain terpene synthase (TPS) genes^10^.

*TPS* genes encode enzymes that catalyze the conversion of oligoprenyl diphosphates of various chain lengths to terpene hydrocarbons or alcohols of enormous structural diversity^11,12^. The finding that dictyostelids contain *TPS* genes is significant in a number of ways. First, it indicates a wider distribution of *TPS* genes, which were previously observed only in plants and fungi among the eukaryotes. Second, it raises an intriguing question about the function of *TPS* genes in dictyostelids. In plants, where *TPS* genes are best studied, they are responsible for making terpenes that are involved in many processes of plant interactions with the environment as a chemical language^13^. Our previous study showed that *TPS* genes are absent in several species of solitary amoeba that are closely related to dictyostelids^10^. It is therefore tempting to speculate that *TPS* genes contribute to the multicellular aspect of the dictyostelids’ unique life style. *Dictyostelium discoideum* has been used as a model species for functional characterization of *TPS* genes. This species contains 9 *TPS* genes, all of which were shown to be expressed and active for the *in vitro* production of sesquiterpenes, while some of them also produce monoterpenes and diterpenes. Many of the identified *in vitro* terpene products were also released as volatile compounds from *D*. *discoideum* during multicellular development^10^.

The dictyostelids are a monophyletic clade that can be subdivided into four taxonomic groups using molecular phylogeny^14,15^. These four groups display enormous diversity in morphology and development^16^. In our previous study, *TPS* genes were found in five species of dictyostelids and the number of *TPS* genes in these species ranges from 1 to 19^10^. Such variation in the number of *TPS* genes poses intriguing questions: do paralogs in each species have similar or different catalytic functions? do paralogs and orthologs have similar or diverging expression patterns? how do the *in vitro* enzyme activities and gene expression patterns determine the chemical diversity of volatile terpenes produced by each dictyostelid species? what biological functions do these *TPS* genes and their terpene products have? Towards answering these questions, in this report, we investigate the functional evolution of the *TPS* family in dictyostelids using *D*. *purpureum* as the main model species.

## Results and Discussion

### Evolutionary relatedness of *TPS* genes from six species of dictyostelids

**Figure 1:**
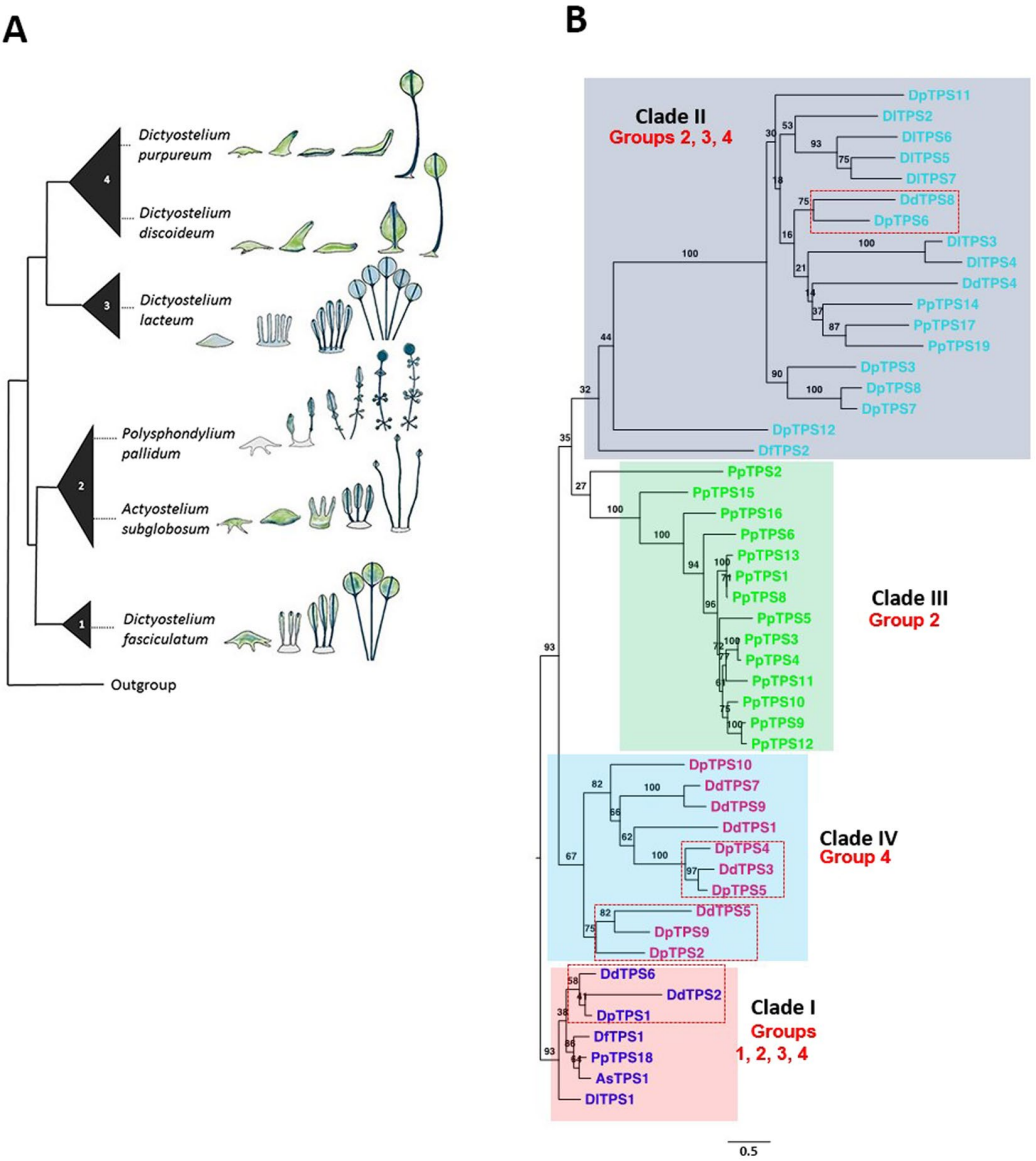
Phylogeny of six species of dictyostelid social amoeba and their terpene synthase genes. (**A**) Phylogeny of six species of dictyostelid social amoeba representing the four taxonomic groups adapted from^3^. Cartoons show the development patterns of the six species. (**B**) Phylogenetic tree of dictyostelid TPSs reconstructed using the maximum likelihood method. Protein sequences of 50 *TPS* genes include 19, 12, 9, 7, 2 and, 1 from *P*. *pallidum*, *D*. *purpureum*, *D*. *discoideum*, *D*. *lacteum*, *D*. *fasciculatum* and, *A*. *subglobosum*, respectively. These genes formed four groups, highlighted with skyblue, light pink, light green, and grey. Ten genes from *D*. *discoideum* and *D*. *purpureum* occurred in the skyblue group. Representative genes from each species *PpTPS18*, *DdTPS6*, *DpTPS1*, *DfTPS1*, *DlTPS1*, and *AsTPS1* were found in the light pink group. Fourteen genes from *P*. *pallidum* made up the light green group. Five genes from *D*. *purpureum* and six genes from *D*. *lacteum* were present in the grey group.

To conduct a comprehensive comparative analysis of the dictyostelid *TPS* gene family, six representative species were selected: *D*. *fasciculatum*, *Actyostelium subglobosum*, *Polysphondylium pallidum*, *D*. *lacteum*, *D*. *purpureum*, and *D*. *discoideum*. There were two main justifications for this selection. First, these six species cover all major taxonomic groups of dictyostelids, belonging to groups 1, 2, 2, 3, 4, and 4, respectively (Fig. 1A^14^). These species exhibit large or small variations in development and morphology. *D*. *fasciculatum*, *A*. *subglobosum*, and *P*. *pallidum* form fruiting bodies from the location of the aggregate and their fruiting bodies have branches, while *D*. *purpureum* and *D*. *discoideum* form slugs that migrate prior to culmination. Second, the genomes of the six species have been fully sequenced^3,5,6,8,17^, allowing for an in-depth analysis of their *TPS* genes. As reported in our previous study, the genomes of the first five species contain 2 (*D*. *fasciculatum*), 1 (*A*. *subglobosum*), 19 (*P*. *pallidum*), 12 (*D*. *purpureum*), and 9 *TPS* genes (*D*. *discoideum*)^10^. The genome sequence of *D*. *lacteum* was recently reported^3^ and we identified 7 *TPS* genes there (Table S1).

To understand their evolutionary relatedness, TPSs from the six dictyostelids species were subjected to a phylogenetic analysis. Four clades (I to IV) could be recognized (Fig. 1B). The phylogeny of clade I is generally consistent with the phylogeny of the six species determined using other genes^18^. All species contain one gene in this clade except for *D*. *discoideum*, which contains two genes *DdTPS6* and *DdTPS2*. While *DdTPS6* appears to be the ortholog, *DdTPS2* most likely resulted from a relatively recent gene duplication of the DdTPS6/2 ancestor that occurred only in *D*. *discoideum* after the split of *D*. *purpureum* and *D*. *discoideum* in group 4.

The split of the two branches of dictyostelids (groups 1 and 2 being one branch and groups 3 and 4 being the other branch) was estimated to have occurred between 600 and 400 million years ago (MYA)^6,8^, indicating that dictyostelidal TPSs are ancient. Clade II contains *TPS* genes from three taxonomic groups, group 2, group 3 and group 4, suggesting a possible loss of *TPS* genes in two species in branch I (*D*. *fasciculatum* and *A*. *subglobosum*). Clade III is specific to *P*. *pallidum*, implying extensive gene family expansion in this species after its divergence from the main branch. Clade IV is group 4-specific, containing only *TPS* genes from *D*. *purpureum* and *D*. *discoideum*, implying the emergence of the genes after the split of the *D*. *purpureum* and *D*. *discoideum* common ancestor from group 3. Between the 12 *TPS* genes from *D*. *purpureum* and 9 *TPS* genes from *D*. *discoideum*, two species in group 4, four putative orthologous groups could be identified (Fig. 1B).

### Catalytic activities of TPSs from *D*. *purpureum*

Prior to this study, *D*. *discoideum* was the only species of dictyostelids in which *TPS* genes have been functionally characterized^10^. To gain insights into the functional evolution of dictyostelid TPSs through comparative analysis, in this study, *D*. *purpureum* was selected as a model species for TPS functional characterization. Both *D*. *purpureum* and *D*. *discoideum* belong to group 4 and are most closely related among the six species examined in this work (Fig. 1A), despite having a large evolutionary distance of 400–300 million years^8^. *D*. *purpureum* and *D*. *discoideum* have been used as a pair of model species for comparative genomics^8^, comparative transcriptome analyses^19^ and comparative biological studies^20^ of dictyostelids.

**Figure 2:**
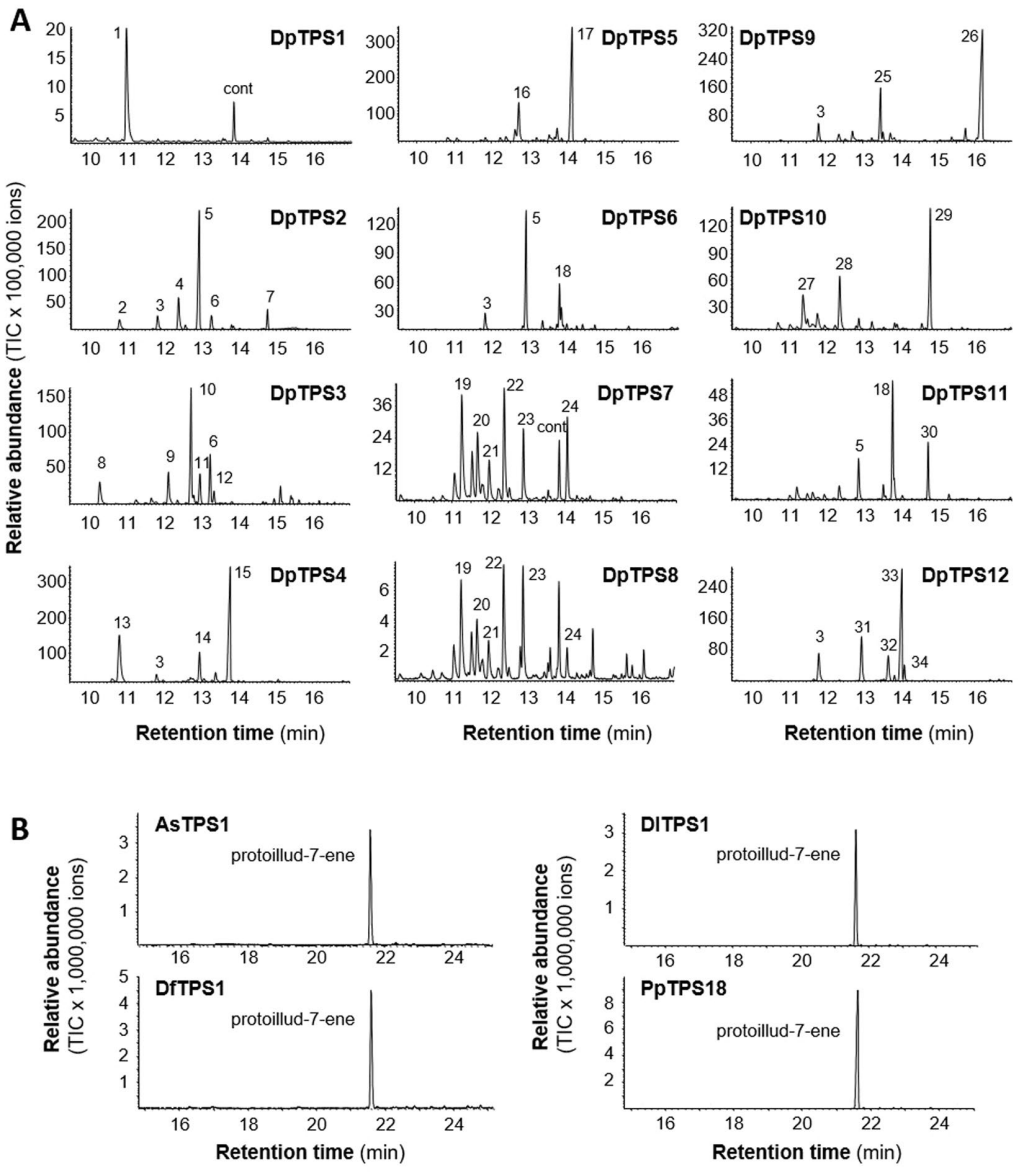
Sesquiterpene synthase activities of recombinant terpene synthases from Dictyostelids. (**A**) Sesquiterpene synthase activities of 12 TPSs from *D*. *purpureum*. *DpTPS* genes were expressed in *E*. *coli* individually, and crude proteins were isolated and their activities were analyzed with farnesyl diphosphate as substrate. 1, protoillud-7-ene; 2, δ-elemene; 3, β-elemene; 4, (*E*)-β-caryophyllene; 5, (*E*)-β-farnesene; 6, 9-*epi*-(*E*)-caryophyllene; 7–9, unidentified sesquiterpenes (STs); 10, CAS 137235-51-9; 11; α-neoclovene; 12, β-neoclovene; 13, bicycloelemene; 14–17; unidentified STs; 18, (*E*,*E*)-α-farnesene; 19–24, unidentified STs; 25, germacrene D; 26, unidentified oxygenated ST; 27–31, unidentified STs; 32, valencene; 33, α-selinene; 34, 7-*epi*-α-selinene; cont, contamination. (**B**) Sesquiterpene synthase activities of recombinant TPSs in the cluster of DpTPS1 orthologs identified by phylogenetic analysis (Fig. 1). AsTPS1 from *A*. *subglobosum*, DfTPS1 from *D*. *fasciculatum*, PpTPS18 from *P*. *pallidum* and DlTPS1 from *D*. *lacteum*. The sesquiterpene products from all four enzymes were identified to be protoillud-7-ene.

*D*. *purpureum* contains 12 *TPS* genes (designated *DpTPS1-12*), in contrast to 9 *TPS* genes in *D*. *discoideum*. Full-length cDNAs for all 12 *TPS* genes from *D*. *purpureum* were cloned and heterologously expressed in *Escherichia coli*. Because most DdTPSs function as sesquiterpene synthases^10^, for comparison, individual recombinant DpTPSs were first tested with farnesyl diphosphate, the substrate for sesquiterpene synthases. All 12 DpTPSs were capable of producing sesquiterpene hydrocarbons or alcohols, indicating that the genes encode *bona fide* TPS enzymes (Fig. 2A). While DpTPS1 produced a sole compound, protoillud-7-ene, all the other 11 DpTPSs catalyzed the formation of complex sesquiterpene mixtures. The product spectra of DpTPS2 and DpTPS6 were dominated by the acyclic compound (*E*)-β-farnesene. DpTPS11 and DpTPS12 produced (*E*,*E*)-α-farnesene and α-selinene, respectively, as main products. The other enzymes formed mixtures of mainly unidentified sesquiterpene hydrocarbons or oxygenated sesquiterpenes.

Beside their sesquiterpene synthase activity, DpTPSs except DpTPS1 and DpTPS10 also exhibited monoterpene synthase activity when provided with geranyl diphosphate as substrate. While DpTPS3, DpTPS4, DpTPS6, DpTPS7, DpTPS8, DpTPS11, and DpTPS12 formed linalool as the major monoterpene product, DpTPS2 and DpTPS9 produced β-myrcene and DpTPS5 produced (*Z*)-β-ocimene as major product (Fig. S1). Seven DpTPSs accepted also geranylgeranyl diphosphate as substrate and catalyzed the formation of different unidentified diterpene alcohols (Fig. S2).

Based on the phylogenetic analysis, *D*. *purpureum* DpTPS1, DpTPS6, DpTPS2/9, and DpTPS4/5 were inferred to be orthologous to *D*. *discoideum* DdTPS6, DdTPS8, DdTPS5, and DdTPS3, respectively (Fig. 1B). The complete biochemical characterization of the DdTPS family (Fig. 2) and the DpTPS family^10,21^ enabled the functional comparison of these putative orthologs (Fig. S3). The first orthologous pair, DpTPS1 and DdTPS6, exhibited identical activities, producing a single sesquiterpene protoillud-7-ene, whereas the second orthologous pair, DpTPS6 and DdTPS8, exhibited distinct activities. The major product of DpTPS6 is (*E*)-β-farnesene, whereas the major product of DdTPS8 is an unidentified sesquiterpene. (*E*,*E*)-α-Farnesene is a common product of DpTPS7 and DdTPS8. The third putative pair of orthologs contains two genes from *D*. *purpureum*, DpTPS2 and DpTPS9. These two have completely distinct catalytic activities. The product profile of DdTPS5 is more similar to that of DpTPS2, with (*E*)-β-farnesene being the most abundant product for both enzymes. The fourth putative ortholog pair also contains two genes from *D*. *purpureum*, DpTPS4 and DpTPS5. While the catalytic activities of DpTPS4 and DpTPS5 are completely different, the two major products of DpTPS5 (unidentified sesquiterpenes) have the same mass spectra as the two unidentified major products of DdTPS3.

### Orthologous TPSs of clade I across the six dictyostelid species have the same catalytic activities

The orthologous pair of *DpTPS1* and *DdTPS6* belongs to clade I, and these enzymes have apparent orthologs in the other four species: DfTPS1 from *D*. *fasciculatum*, AsTPS1 from *A*. *subglobosum*, PpTPS18 from *P*. *pallidum*, and DlTPS1 from *D*. *lacteum* (Fig. 1B). The observation that DpTPS1 and DdTPS6 have the same catalytic activity prompted us to ask whether the other orthologs have the same catalytic activities as well. To answer this question, the full-length cDNAs for *DfTPS1*, *AsTPS1*, *PpTPS18* and *DlTPS1* were cloned from the respective species and expressed in *E*. *coli* to produce recombinant proteins. Testing with farnesyl diphosphate, all four TPSs catalyzed the formation of the same sesquiterpene protoillud-7-ene, exhibiting the same catalytic activity as DpTPS1 and DdTPS6 (Fig. 2B). This indicates that the sole orthologous group among all the six species has an identical biochemical function despite more than 600 million years of divergence^6^. Such functional conservation is rare among TPSs from plants, fungi and bacteria.

### Expression patterns of *TPS* genes during development of *D*. *purpureum* and comparative analysis

**Figure 3:**
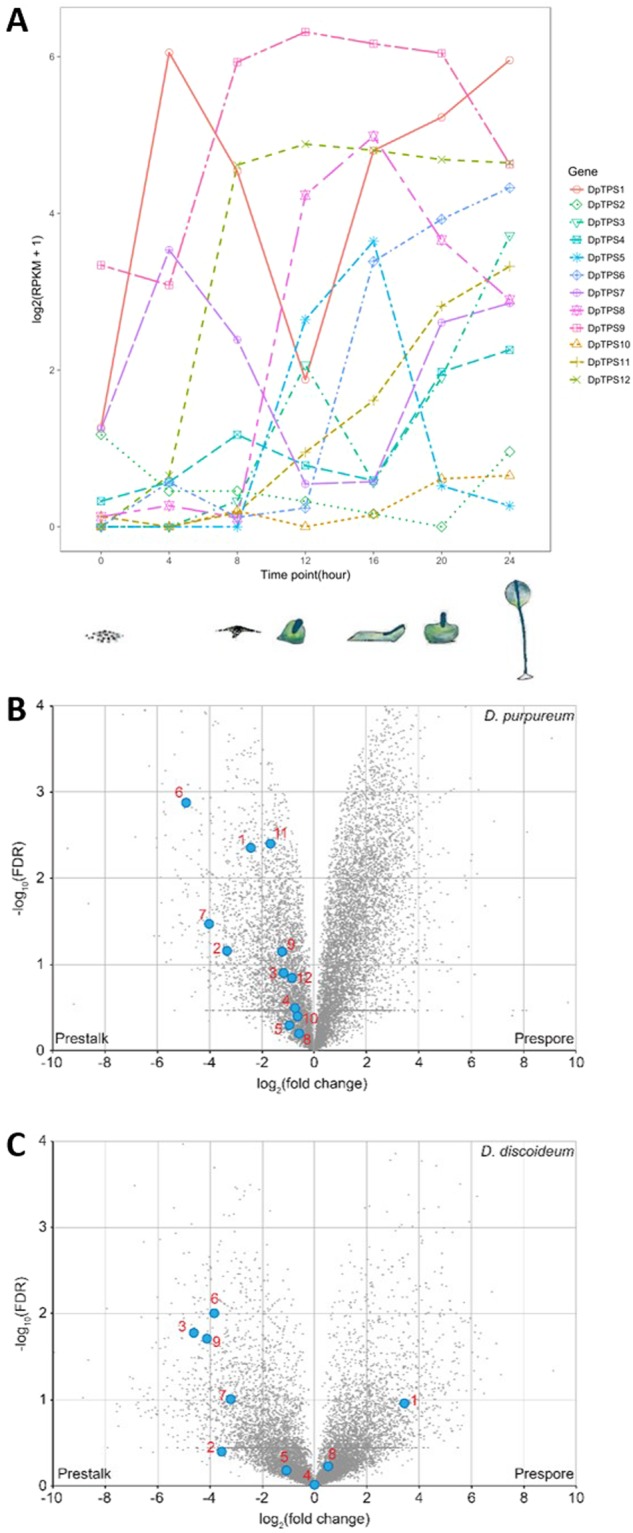
Expression of terpene synthase genes during dictyostelid development. (**A**) Expression of *DpTPS* genes during 24 hour development. Expression of *DpTPS* genes was measured by RPKM (reads per kilobase per million sequenced reads) based on RNA-Seq data produced from web-based interface program (http://dictyexpress.biolab.si/) and then displayed on a log2(RPKM+1) scale in this line plot. The cartoons depict six stages of multicellular development: vegetative, individual cells (0 h), streaming (8 h), loose aggregate (10 h), slug (16 h), Mexican hat (20 h), and fruiting bodies (24 h). (**B**) Expression of *D*. *purpureum TPS* genes in prestalk and prespore cells. The numbers 1 to 12 correspond to *DpTPS1* to *DpTPS12*. (**C**) Expression of *DdTPS* genes from *D*. *discoideum* in prestalk and prespore cells. The numbers 1 to 9 correspond to *DdTPS1* to *DdTPS9*.

The multicellular development program in *D*. *purpureum* is initiated upon starvation. The 24-hour developmental process can be broadly divided into several stages: streaming, loose aggregate, mound, Mexican hat, and fruiting body (Fig. 1A). The expression of the *DpTPS* genes at seven time points during the 24 h development with 4 h intervals was extracted from a previous transcriptome analysis^19^ and presented in Fig. 3A.

All twelve *TPS* genes exhibited moderate levels of mRNA abundance and vast changes during the 24-hour course of the developmental process. Based on the mRNA abundance at the time of peak expression, the 12 genes can be categorized into three groups (Fig. 3A): high-abundance genes included *DpTPS1*, *DpTPS8*, *DpTPS9* and *DpTPS12*, while medium-abundance genes were represented by *DpTPS3*, *DpTPS5*, *DpTPS6* and *DpTPS7*, and low-abundance genes were *DpTPS2*, *DpTPS4*, *DpTPS10* and *DpTSP11*. Furthermore, different time points for the highest expression levels of the *DpTPS* genes were observed. *DpTPS2* was the only gene whose highest abundance levels occurred during vegetative growth (0 h). For all other genes, peak abundance occurred at various times during development. *DpTPS1* and *DpTPS7* were the two genes whose peak abundance happened at a time point during the first half of development. The highest expression of *DpTPS9* and *DpTPS12* occurred at the half point of development (12 h), while the peak abundance of the rest of the *DpTPS* genes was recorded during the second half of development (Fig. 3A).

The expression of orthologous pairs/groups between *D*. *purpureum* and *D*. *discoideum* was also compared (Fig. S4). It appeared that *DpTPS9* and *DdTPS5* had similar patterns of expression, and the same applied to *DpTPS1* and *DdTPS6*. There were similarities between the expression patterns of *DpTPS5* and *DdTPS3*, although the abundance of *DdTPS5* was much higher.

During multicellular development, cells of *Dictyostelium* amoebae differentiate into two types: prestalk cells and prespore cells, which will eventually develop into the stalk and spores of the fruiting body^22^. All 12 *DpTPS* genes in *D*. *purpureum* were enriched in prestalk cells (Fig. 3B). This pattern is different from that of *DdTPS* genes in *D*. *discoideum*. While the majority of *DdTPS* genes also showed preferential expression in prestalk cells, two of them, *DdTPS1* and *DdTPS8*, have higher expression in prespore cells (Fig. 3C). This is an intriguing observation, but its significance cannot be evaluated until we understand the biological functions of the terpenes produced by these gene products.

### Emission of volatile terpenes during development of *D*. *purpureum* and comparative analysis

Most *DpTPS* genes showed dynamic expression during multicellular development, suggesting that they may have a function during this process. In addition, all of the DpTPSs were active in producing sesquiterpenes and seven of them were active in producing monoterpenes in the *in vitro* enzyme assays (Fig. 2A). Since sesquiterpenes and monoterpenes are volatiles, we performed volatile profiling of *D*. *purpureum* culture during 24 h development. Volatile collections were performed in every four hours.

**Figure 4:**
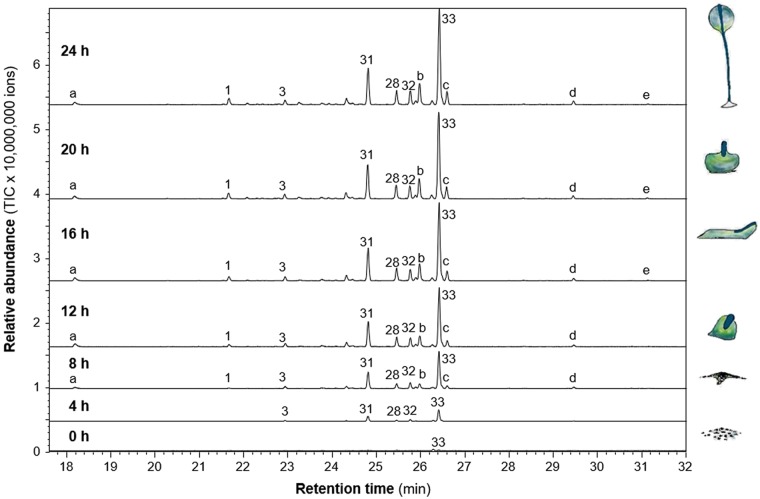
Volatile profiles of *Dictyostelium purpureum* culture during development. Volatile emissions during development of *D*. *purpureum* were collected by SPME and analyzed using GC-MS. All peaks labeled with a number are sesquiterpenes and the numbers correspond to the numbers of peaks in Figs 2 and 3. 1, protoillud-7-ene*; 3, β-elemene*; 28, germacrene D; 31, unidentified sesquiterpene hydrocarbon; 32, valencene; 33, α-selinene. Letters indicate compounds that are not found in the product spectra of DpTPS. “a”, unidentified compound; “b”, widdrol; “c”, unidentified oxygenated sesquiterpene; “d”, allo-hedycaryol; “e”, unidentified oxygenated terpene. Compounds marked with asterisks (*) were identified using authentic standards. The cartoons depict six stages of multicellular development: vegetative, individual cells (0 h), streaming (8 h), loose aggregate (10 h), slug (16 h), Mexican hat (20 h) and fruiting bodies (24 h).

During this time course experiment a mixture of eight volatile sesquiterpenes was detected (Fig. 4), most of them were identified as the products of DpTPS1, DpTPS9 and DpTPS12. The predominant sesquiterpene was α-selinene, the major product of DpTPS12. Besides α-selinene, β-elemene, an unidentified sesquiterpene (peak#31) and valencene were minor products produced by DpTPS12. From time point 4 to 24 hour, emission of these four sesquiterpenes produced by DpTPS12 increased with time, and reached the highest levels at 24 h. Protoillud-7-ene and germacrene D were produced by DpTPS1 and DpTPS9, respectively.

*D*. *discoideum* also released a mixture of volatile terpenes, including nine sesquiterpenes, one monoterpene and one diterpene^10^. In contrast to *D*. *purpureum* in which three *DpTPS* genes are main contributors of volatile production, the terpene products of all DdTPSs except DdTPS8 could be validated by the detection of DdTPS products in the volatile bouquet of *D*. *discoideum*^10^. Among all the volatile terpenes emitted from the two species, only one terpene is common: protoillud-7-ene, which is the product of the ortholog pair DpTPS1 and DdTPS6 in the clade 1 (Fig. 1B). In *D*. *purpureum*, α-selinene is the most abundant terpene at any time throughout multicellular development. In contrast, in *D*. *discoideum* the most abundant terpene before 24 hours is different from that at 24 h during development.

## Concluding Remarks

This study has led to new insights into the diversity and functional evolution of the *TPS* gene family in dictyostelid social amoebae. Through phylogenetic analysis, a conserved clade of *TPS* genes from the six species of social amoebae was identified (Fig. 1B), implying a common evolutionary origin of dictyostelid *TPS* genes. The variation in the number of *TPS* genes among the six species suggests group- or species-specific expansion of *TPS* genes through gene duplication. At the biochemical level, individual orthologs in this conserved clade encode enzymes of the same catalytic activity (Fig. 2B), while the paralogs in *D*. *purpureum* (Fig. 2A) as well as those in *D*. *discordeum*^10^ exhibit divergent catalytic functions. While essentially nothing is known about the biological function of terpenoids in dictyostelids, this family of metabolites is involved in diverse biological processes in other organisms. In plants, for example, some terpenoids function as phytohormones critical for regulating growth and development and many others are involved in mediating plant-environment interactions^13^. In analogy, protoillud-7-ene, the product of *DpTPS6* and its orthologs, may play a conserved, critical role, such as in regulating multicellular development, while the products of other *TPS* genes may have group- or species-adaptive functions, such as in chemotaxis^23^, defense^24^, or attracting beneficial organisms^25^. Such functions have been proposed for terpenoids as well as other types of secondary metabolites made by dictyostelid social amoebae^26^. Knowledge of the catalytic activities of individual TPSs and their contribution to the biosynthesis of volatile terpenoids during multicellular development in *D*. *purpureum* and *D*. *discoideum* now enables future researchers to elucidate the specific role of individual *TPS* genes and their terpenoid products. For this endeavor, mutant strains with disrupted *TPS* genes that could be produced using restriction enzyme-mediated integration (REMI) mutagenesis^27^ or recently reported CRISPR/Cas9 technology for *Dictyostelium*^28^ will be particularly useful.

## Materials and Methods

### Experimental organisms

*D*. *purpureum* DpAX1 (DBS0308472), *Acytostelium subglobosum* LB1 (DBS0235452), *D*. *fasciculatum* SH3 (DBS0235810), *D*. *lacteum* (DBS0235831) and *Polysphondylium pallidum* PN500 (DBS0236808) were obtained from Dicty Stock Center (http://dictybase.org/). Five fruiting bodies of *D*. *purpureum* DpAX1 were added into 300 µl liquid culture of *Klebsiella pneumonia*, mixed well, and spread onto SM agar plate (http://dictybase.org/). Five Fruiting bodies of *A*. *subglobosum* LB1, *D*. *fasciculatum* SH3, *D*. *lacteum* and *P*. *pallidum* PN500 were mixed with *E*. *coli* B/r stain, and spread onto LP agar plates (http://dictybase.org/). The plates were incubated at 22 °C in the dark.

### Sequence and phylogenetic analysis

Amino acid sequence dataset of *D*. *lacteum* (http://sacgb.leibniz-fli.de) was downloaded and searched against Pfam-A database locally using HMMER 3.0. Putative terpene synthase genes were identified using a HMM profile Terpene_synth_C (PF03936) with an e-value of 1*e*^−2^. Multiple sequences were aligned using MAFFT(L-INS-i). The maximum likelihood phylogenetic trees were built using RAxML under the LG + G + F model with 1000 bootstrap replicates.

### Full-length cDNA cloning

Social amoeba tissues at the stage of fruiting body were collected and homogenized using TissueLyser II according to manufacturer’s manual (https://www.qiagen.com). Total RNA was isolated using RNeasy Mini kit following manufacturer’s protocol (https://www.qiagen.com). cDNA was synthesized using First Strand Synthesis Kit according to manufacturer’s protocol (http://www.gelifesciences.com). Full-length cDNA of individual *TPS* genes was amplified with gene specific primers (Table S2), cloned into vector pEXP5-CT/TOPO (https://www.thermofisher.com), and fully sequenced.

### Terpene synthase enzyme assays

Individual social amoeba *TPS* genes in the pEXP5-CT/TOPO protein expression vector were transformed into *E*. *coli* Bl21(DE3) for heterologous protein expression. Crude protein extracts were desalted into assay buffer (10 mM Tris-HCl, pH 7.5, 1 mM dithiothreitol, 10% (v/v) glycerol) before enzyme assays. The catalytic activity of each social amoeba TPS was determined in assays containing 50 μl of the crude protein and 50 µl of assay buffer with 10 μM substrate (geranyl diphosphate, farnesyl diphosphate or geranylgeranyl diphosphate), 10 mM MgCl_2_ and 0.05 mM MnCl_2_. A solid phase microextraction (SPME) fiber consisting of 100 µm polydimethylsiloxane (SUPELCO, Belafonte, PA, USA) was inserted into the headspace of the assay vial. After incubation at 30 °C for 1 h, the SPME fiber was withdrawn from the vial and then inserted directly into the injector of the gas chromatograph. For the substrate geranylgeranyl diphosphate, assays were overlayed with 100 µl hexane. After extraction by vortexing, 2 µl of the organic phase was analyzed by GC/MS. A Hewlett-Packard model 6890 gas chromatograph coupled with a Hewlett-Packard model 5973 mass spectrometer with a quadrupole mass selective detector was employed for product identification. Each assay was repeated at least three times and the results were found to be consistent.

### Expression analysis of terpene synthase genes in *D*. *purpureum* and *D*. *discoideum*

The expression data of individual *TPS* genes in *D*. *purpureum* and *D*. discoideum was obtained from the dataset reported previously^29^ and profiled using the dictyExpress web interface (https://dictyexpressresearch.bcm.edu/landing/).

### Volatile profiling

*D*. *purpureum* spores and freshly grown *K*. *pneumonia* were mixed and spread onto SM agar plates to initiate culture. Volatiles of *D*. *purpureum* cultures during 24-hour multicellular development were collected at seven time points (every 4 hours) using SPME and analyzed using GC-MS as previously reported^10^.

## Electronic supplementary material

Supplementary information

## Data Availability

Sequences of functionally characterized terpenes synthase genes from Dictyostelid social amoebae are available on GenBank: MG262459-MG262475.

## References

[1] Schaap P (2016). Evolution of developmental signalling in Dictyostelid social amoebas. Curr Opin Genet Dev.

[2] Du Q, Kawabe Y, Schilde C, Chen ZH, Schaap P (2015). The Evolution of Aggregative Multicellularity and Cell-Cell Communication in the Dictyostelia. J Mol Biol.

[3] Glockner G (2016). The multicellularity genes of dictyostelid social amoebas. Nature Commun.

[4] Schilde C (2016). A set of genes conserved in sequence and expression traces back the establishment of multicellularity in social amoebae. BMC Genomics.

[5] Eichinger L (2005). The genome of the social amoeba *Dictyostelium discoideum*. Nature.

[6] Heidel AJ (2011). Phylogeny-wide analysis of social amoeba genomes highlights ancient origins for complex intercellular communication. Genome Res.

[7] Zucko J (2007). Polyketide synthase genes and the natural products potential of *Dictyostelium discoideum*. Bioinformatics.

[8] Sucgang R (2011). Comparative genomics of the social amoebae *Dictyostelium discoideum* and *Dictyostelium purpureum*. Genome Biol.

[9] Ghosh R (2008). Dissecting the functional role of polyketide synthases in *Dictyostelium discoideum*: biosynthesis of the differentiation regulating factor 4-methyl-5-pentylbenzene-1,3-diol. J Biol Chem.

[10] Chen X (2016). Terpene synthase genes in eukaryotes beyond plants and fungi: Occurrence in social amoebae. Proc Natl Acad Sci USA.

[11] Chen F, Tholl D, Bohlmann J, Pichersky E (2011). The family of terpene synthases in plants: a mid-size family of genes for specialized metabolism that is highly diversified throughout the kingdom. Plant J.

[12] Christianson DW (2008). Unearthing the roots of the terpenome. Curr Opin Chem Biol.

[13] Gershenzon J, Dudareva N (2007). The function of terpene natural products in the natural world. Nat Chem Biol.

[14] Schaap P (2006). Molecular phylogeny and evolution of morphology in the social amoebas. Science.

[15] Romeralo M, Cavender JC, Landolt JC, Stephenson SL, Baldauf SL (2011). An expanded phylogeny of social amoebas (Dictyostelia) shows increasing diversity and new morphological patterns. BMC Evol Biol.

[16] Schaap P (2007). Evolution of size and pattern in the social amoebas. BioEssays.

[17] Urushihara H (2015). Comparative genome and transcriptome analyses of the social amoeba *Acytostelium subglobosum* that accomplishes multicellular development without germ-soma differentiation. BMC Genomics.

[18] Singh R, Schilde C, Schaap P (2016). A core phylogeny of Dictyostelia inferred from genomes representative of the eight major and minor taxonomic divisions of the group. BMC Evol Biol.

[19] Parikh A (2010). Conserved developmental transcriptomes in evolutionarily divergent species. Genome Biol.

[20] Jack CN (2008). Segregate or cooperate- a study of the interaction between two species of *Dictyostelium*. BMC Evol Biol.

[21] Rabe P (2016). Terpene Cyclases from Social Amoebae. Angew Chem Int Ed Engl.

[22] Jermyn KA, Duffy KT, Williams JG (1989). A new anatomy of the prestalk zone in *Dictyostelium*. Nature.

[23] King JS, Insall RH (2009). Chemotaxis: finding the way forward with *Dictyostelium*. Trends Cell Biol.

[24] Kessin RH, Gundersen GG, Zaydfudim V, Grimson M (1996). How cellular slime molds evade nematodes. Proc Natl Acad Sci USA.

[25] Smith J, Queller DC, Strassmann JE (2014). Fruiting bodies of the social amoeba *Dictyostelium discoideum* increase spore transport by *Drosophila*. BMC Evol Biol.

[26] Barnett R, Stallforth P (2018). Natural Products from Social Amoebae. Chemistry.

[27] Kuspa A (2006). Restriction enzyme-mediated integration (REMI) mutagenesis. Methods Mol Biol.

[28] Sekine R, Kawata T, Muramoto T (2018). CRISPR/Cas9 mediated targeting of multiple genes in *Dictyostelium*. Sci Rep.

[29] Rot G (2009). dictyExpress: a *Dictyostelium discoideum* gene expression database with an explorative data analysis web-based interface. BMC Bioinformatics.

