# Supplementary material for: Diversity and Functional Evolution of Terpene Synthases in Dictyostelid Social Amoebae
Source: Sci Rep. 2018 Sep 25;8:14361. doi: 10.1038/s41598-018-32639-0 (PMC6156593; doi:10.1038/s41598-018-32639-0)
Supplement: Supplementary file 1 — Supplementary information [file 41598_2018_32639_MOESM1_ESM.pdf]

Diversity and Functional Evolution of Terpene Synthases in Dictyostelid Social Amoebae

Xinlu Chen, Tobias G. Köllner, Gad Shaulsky, Qidong Jia, Jeroen S. Dickschat, Jonathan Gershenzon & Feng Chen

Table S1. List of terpene synthase genes from *Dictyostelium lacteum*

Gene name	Gene ID	location	Protein size	Number of introns
DlTPS1	DLAC_07096	LODT01000031.1:625710-626848	354	1
DlTPS2	DLAC_10965	LODT01000051.1:c207457-206420	345	0
DlTPS3	DLAC_05221	LODT01000025.1:c629567-628551	338	0
DlTPS4	DLAC_02702	LODT01000013.1:c532509-531535	324	0
DlTPS5	DLAC_05826	LODT01000028.1:1030493-1031416	308	0
DlTPS6		LODT01000028.1:1031498-1032448	317	0
DlTPS7		LODT01000028.1:1032728-1033668	279	1

Table S2 Primers used for full-length cDNA cloning

Gene Name	Primers	Sequences
	Forward	5'-ATGTCATTATCATTAAGCGATATTAAATTTC-3'
DpTPS1	Reverse	5'-TTAATTTATAATTTCTCTGGTGGTAAAAATG-3'
	Forward	5'-ATGAATTTAACACTTAAAGATTTTCATG-3'
DpTPS2	Reverse	5'-TTATAGTTCATTATTGATTAATTTATTATC-3'
	Forward	5'-ATGCAAAAAGAAATTTATTATACATTTGAC-3'
DpTPS3	Reverse	5'-TTAATGATTTAAATTATAACGTGGGTAG-3'
	Forward	5'-ATGAATAGCTTAGCAAACAATGGC-3'
DpTPS4	Reverse	5'-TTATATCCAAAATATATCAGGTGTTAAAATTG-3'
	Forward	5'-ATGCAAGAAAAACCAAATCTTTTAAG-3'
DpTPS5	Reverse	5'-TTAATAAAGATTTGAATTTAAGATCTTTTC-3'
	Forward	5'-ATGAAACAACATATACTTTGGGATTTAAATAAC-3'
DpTPS6	Reverse	5'-TTATTTATAACGGTTACAGATTGAAGAGAC-3'
	Forward	5'-ATGCAAGAAAAATCAAGAGTGTTCAAATG-3'
DpTPS7	Reverse	5'-TTATTTATAATCAGCTTCACAAACAGTG-3'
	Forward	5'-ATGCAAGAAGAAATATTATACAAATGGAATTATG-3'
DpTPS8	Reverse	5'-TTATTCATTAATAATCTTTAATTCAGCTTC-3'
	Forward	5'-ATGTCTCTATCTTTTAAAAATATAGTTTTTC-3'
DpTPS9	Reverse	5'-TTATAAACTGGAGGCATCTATTTCATTATC-3'
	Forward	5'-ATGGAAGCTATAAAACAAAAAAGAAG-3'
DpTPS10	Reverse	5'-TCATCGAAAAAATGTTTTTATGTTGGAC-3'
	Forward	5'-ATGGATATAAATAAACAAAAAACCAAATG-3'
DpTPS11	Reverse	5'-TTAATTATTTTTATTGATATAGTTTTTGTTG-3'
	Forward	5'-ATGTATTCTTTATTTAAAAATATAAAATTTC-3'
DpTPS12	Reverse	5'-CTAACCAAATAATGATTTAGTATTTAAATAAG-3'
	Forward	5'-ATGTCACTATCTTTAAATGATATCAAATTC-3'
AsTPS1	Reverse	5'-TTAAGCCTCACGGAACTTTCTCATTTC-3'
	Forward	5'-ATGTTATCATTAAACGAAATCAAGTTTC-3'
DfTPS1	Reverse	5'-CTAAAGTTTGGACATCAAGTTGCCTTG-3'
	Forward	5'-ATGTCACTGTCACTTAATGATATTAAATTTC-3'
DlTPS1	Reverse	5'-TTATACTCTTTTTCTTTTCTTTAAGTCTC-3'
	Forward	5'-ATGTTGTCCTTAAACGAAATCAAATTTC-3'
PpTPS18	Reverse	5'-TTATAGATAGCTTTTCGAGGGAGGTC-3'

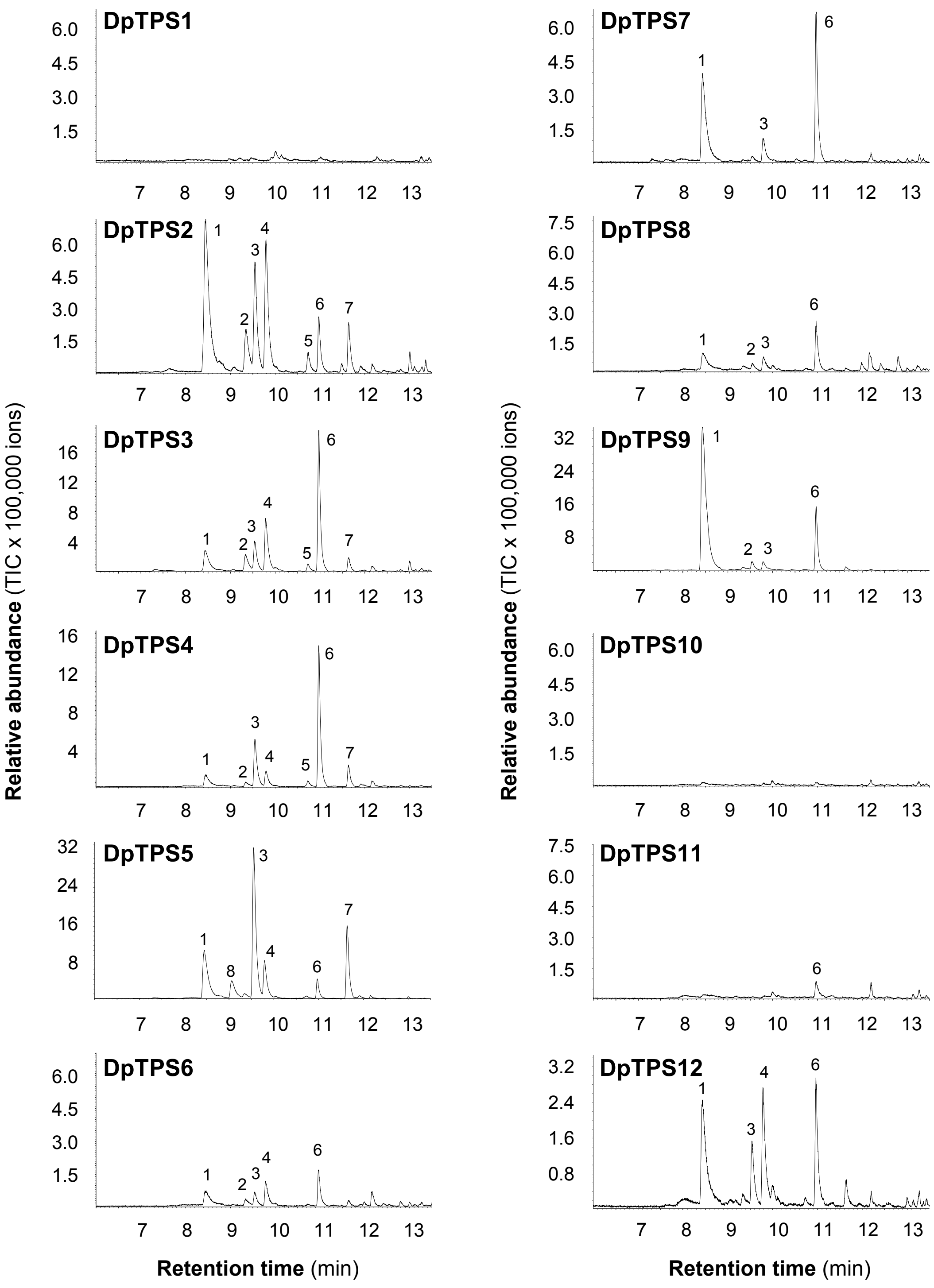

Figure S1. Monoterpene products of *Dictyostelium purpureum* terpene synthases. The *TPS* genes were heterologously expressed in *Escherichi coli* and crude protein extracts were incubated with the substrate GPP in the presence of 10 mM Mg_2Cl as cofactor. The produced monoterpenes were collected from the headspace of the assays using solid-phase microextraction and were analyzed with gas chromatography/mass spectrometry. 1, β-myrcene*; 2, limonene*; 3, (*Z*)-β-ocimene; 4, (*E*)-β-ocimene; 5, terpinolene*; 6, linalool*; 7, neo-allo-ocimene; 8, α-terpinene*.

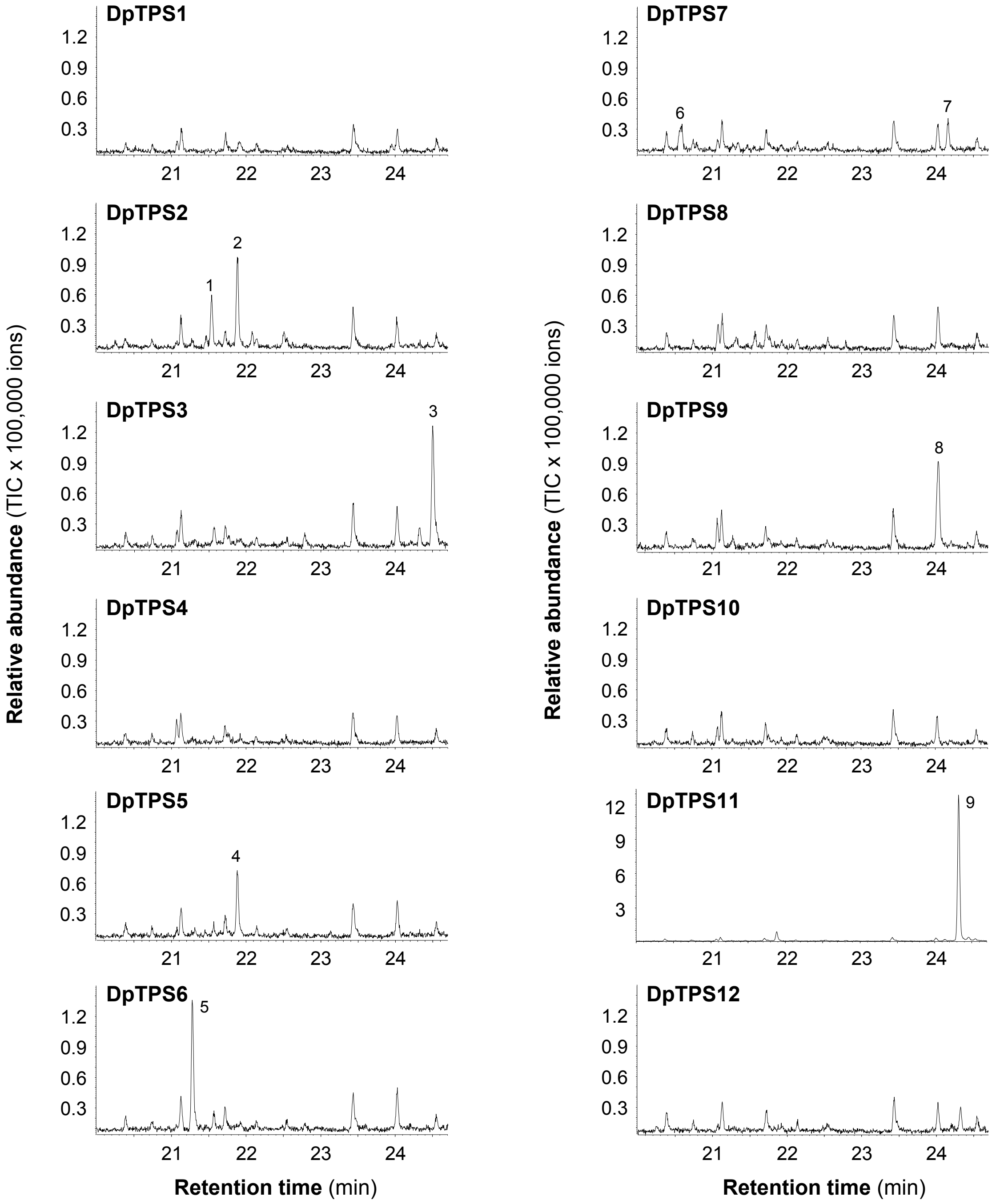

Figure S2. Diterpene products of *Dictyostelium purpureum* terpene synthases. The *TPS* genes were heterologously expressed in *Escherichi coli* and crude protein extracts were incubated with the substrate GGPP in the presence of 10 mM Mg_2Cl as cofactor. The produced diterpenes were extracted from the assays with hexane and were analyzed using gas chromatography/mass spectrometry. 1+2, unidentified DTs; 3+4, unidentified oxygenated DTs; 5+6, unidentified DTs; 7-9, unidentified oxygenated DTs; DT, diterpene hydrocarbon. Non-labeled peaks are contaminations.

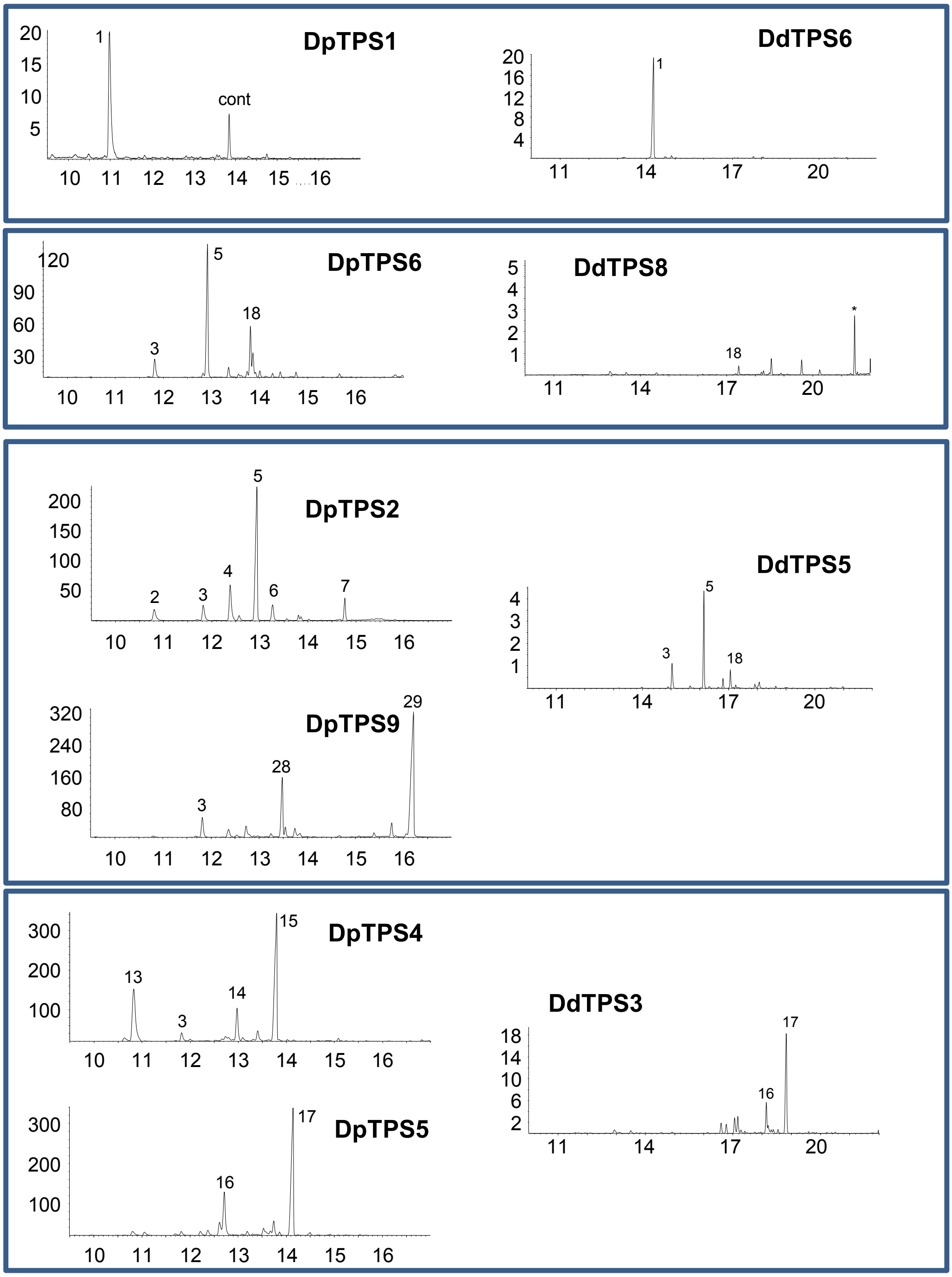

Figure S3. Comparison of sesquiterpene products of four putatvie orthologous groups of terpene synthases from *D. purpureum* (DpTPS) and *D. discoideum* (DdTPS). The compound numbers follow the same scheme as prerented in Figure 2. "*" represents an unidentified sesquiterpene.

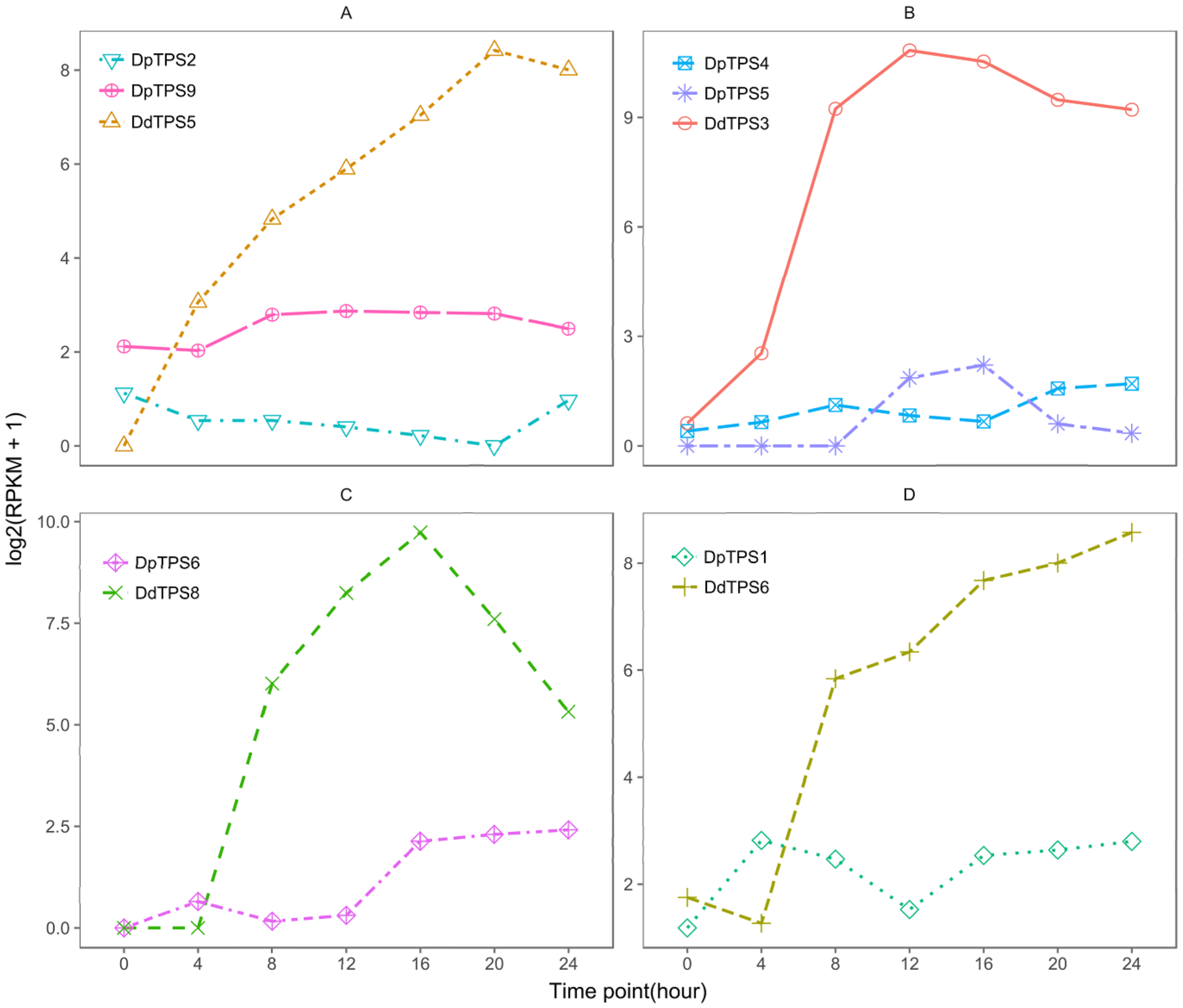

Figure S4. Comparison of expression patterns of foour putative orthologous pairs/groups of terpene synthase genes between *D. purpureum* and *D. discoideum* during 24 hour development. Expression of TPS genes was measured by RPKM (reads per kilobase per million sequenced reads) based on RNA-Seq data produced from web-based interface program (http://dictyexpress.biolab.si/) and then displayed on a log2(RPKM+1) scale in this line plot.
